# Compressive Behavior and Constitutive Model of Austenitic Stainless Steel S30403 in High Strain Range

**DOI:** 10.3390/ma11061023

**Published:** 2018-06-15

**Authors:** Yang Peng, Jiang Chu, Jun Dong

**Affiliations:** College of Civil Engineering, Nanjing Tech University, Nanjing 211816, China; yang.peng@njtech.edu.cn (Y.P.); chujiang@njtech.edu.cn (J.C.)

**Keywords:** compressive behavior in high strain range, stress-strain response, compressive constitutive model, material anisotropy, stainless steel

## Abstract

Material anisotropy for tension and compression is a significant characteristic of austenitic stainless steel compared to carbon steel. Due to limitations during the testing of the restrained jig, the maximum strain value of compressive experiments of austenitic stainless steel is around 2%. This value cannot satisfy the requirements of accurate finite simulation on austenitic stainless steel columns and beams in the high compressive strain range. In this study, a new type of compressive specimen that satisfies the high compressive strain test was designed. The stress-strain response of austenitic stainless steel S30403 (JISCO, Gansu, China) was investigated in the high compressive strain range up to 10%, and constitutive models were compared with the experimental data. It was found that the new type specimen with length-to-diameter ratio of 1:1 can reliably obtain the stress-strain response of austenitic stainless steel S30403 in the high compressive strain range. It was found that the material anisotropy of austenitic stainless steel S30403 is remarkable in the high compressive strain range up to 10%. The strain-hardening curve of the austenitic stainless steel S30403 can be represented by a straight line in the high compressive strain range. Our study also found that the Quach constitutive model accurately describes the two-stage strain-hardening phenomenon in the high compressive strain range up to 10%.

## 1. Introduction

Owing to its easy maintenance and ability to resist corrosion as well as its attractive appearance and excellent structural performance, stainless steel has been increasingly utilized in structural applications. In addition to its low proportional limits, stainless steel is characterized by material anisotropy, round stress-strain behavior, and different constitutive behavior in tensile and compressive loading [1]. Compressive constitutive models for stainless steel have a significant impact on the prediction of the structural behavior of stainless steel columns and beams by numerical simulation [2,3,4,5,6]. The predicted difference in the ultimate axial load of concrete-filled stainless-steel tubular columns is 16.9% when the strain-hardening behavior in tension and compression is ignored [5,6].

As a commonly used material of stainless steel, austenitic stainless steel is initially entirely composed of austenite. The austenite can be transformed into martensite under loading, resulting in a change to the mechanical behavior of austenitic stainless steel. Different constitutive models for austenitic stainless steel have been suggested based on different approaches. Physically based constitutive models have been developed [7,8,9], which can reveal the strain dependence of the kinetics of transformation of the austenitic stainless steel under loading. These models have many material parameters and the identification of these material parameters is time-consuming. However, they are popular in materials engineering research. Two constitutive models proposed by Gardner and Ashraf [10] and Quach et al. [11] are suitable for accurately describing the compressive behavior of austenitic stainless steel [2,3,4,5,6]. These constitutive models were verified by comparing their results with the experimental data from compressive tests published in the literature [10,11,12]. These models are phenomenological; some of the parameters of these models are empirical and obtained by regression. They are popular in engineering applications. However, the reliability of the constitutive models verified by these compressive experimental data in the high strain range is unknown. Constitutive models are therefore needed to verify the experimental data in the high strain range.

Numerous experimental studies of compressive behavior of austenite stainless steel have been performed. Johnson and Kelsen [13] studied the compressive behavior of the 302 and 304 austenitic stainless steel and the maximum strain was less than 1%. They found that austenitic stainless steel was anisotropic. Korvink et al. [14] tested the compressive behavior of ferritic stainless steel 430 and the maximum strain was 0.6%. Gardner et al. [12] tested the compressive behavior of austenitic stainless steel 304, and their results indicated that the compressive stress-strain curves were in agreement with the tensile stress-strain curve with a strain less than 2%. Buchanan et al. [15] tested the additive manufactured austenitic stainless steel 316L under compressive stress, and the maximum strain was 1%. They found that the strength and Young’s modulus of additive manufactured austenitic stainless steel was lower than those of conventional austenitic stainless steel. Overall, the maximum compressive strain was less than 2% of all the compressive experimental data. The specimens buckled under high strain range. To prevent buckling, a lateral support jig was used in the compressive experiments. However, the gap between the lateral support jig and the specimen was limited. Owing to the Poisson effect and volume expansion, the specimen contracted the lateral support jig under high strain, which restricted the deformation of the specimens. Therefore, high compressive strain could not be attained by this test approach. Due to the limitation of these experiments, the constitutive model could not be verified by comparison with the high compressive strain data.

In this study, a new configuration of compressive specimen made with austenitic stainless steel S30403 was designed to be suitable for high compressive strain experiments, and the feasibility of the new specimen was studied. The compressive mechanical properties of austenitic stainless steel S30403 were determined and the material anisotropy under tension and compression was studied. Furthermore, Gardner and Ashraf constitutive model and the Quach model were compared to the compressive test results, and the reliability of these models is discussed.

## 2. Experimental Details

The test material was a low-carbon austenitic stainless steel S30403 (022Cr19Ni10, equivalent to AISI304L and EN 1.4306) that is commonly used in welded structures. The Chinese standard GB/T 4237-2015 [16] provided the chemical composition shown in Table 1 and mechanical properties shown in Table 2.

In compression tests, specimens may exhibit buckling under high strain, including elastic and inelastic buckling. After buckling, the specimens undergo bending or torsional deformation and the gauge stress is unevenly distributed, making the test result ineffective [17]. To avoid buckling in compressive tests, the specimens are generally surrounded by a bracing jig and smeared with lubricating paste—a method that has been widely used [12,15]. However, owing to the limited gap between the bracing jig and the specimen, the stress-strain curve cannot be measured for high strain up to 3% [12,15]. Therefore, this approach cannot obtain the compressive behavior in the high strain range.

**Figure 1:**
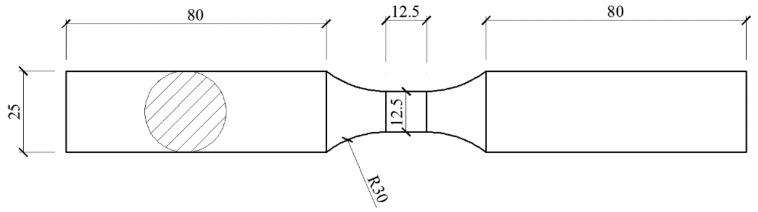
Small-gauge-length specimen.

Another approach is to keep the specimen’s cross-sectional area unchanged, reduce the gauge length of the specimen, and thus reduce the slenderness ratio of the specimen and increase the test specimen’s buckling strength [18]. This approach can obtain compressive behavior in the high strain range. Marsh et al. [19] proposed that the length-to-diameter ratio of the structural steel test specimen (the ratio of the length of the gauge section to the diameter of the gauge section) should be less than or equal to 1.3 to prevent the specimen from buckling. Zhou et al. [20] conducted the structural steel hysteresis test and concluded that the length-to-diameter ratio of the test specimens should be less than or equal to 1.13. Compression test standard GB/T 7314-2005 [17] recommends that the specimen length-to-diameter ratio be between 1 and 2. The tension and compressive properties of stainless steel are significantly different and the strain-hardening phenomenon under compression is higher than that when pulled [21]. The strain hardening of the austenitic stainless steel is higher than that of structural steel [1]. This implies that the force imposed on compressive specimens is higher than that of structural steel specimens when the compressive strain is identical. Austenitic stainless steel specimens require higher buckling strength compared to structural steel specimen when a higher load is imposed on the austenitic stainless steel specimen under the same compressive strain. Considering the variations due to different compressive properties between austenitic stainless steel and structural steel, this test adopted a smaller value than that recommended by Zhou et al. [20]. The length-to-diameter ratio of the specimen was 1:1, according to the lower limit value of the compression test standard GB/T 7314-2005 [17]; the gauge length was 12.5 mm and the gauge diameter was 12.5 mm (Figure 1).

**Figure 2:**
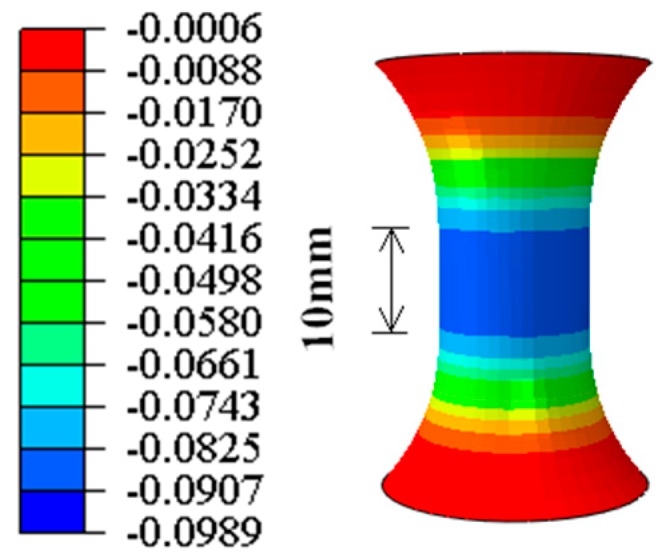
Strain distribution along the gauge length under high compressive strain (*ε* = −10%).

The stress state of the small-gauge-length specimen may be in a triaxial stress state under high strain when the grip section and the fillet are larger than the gauge length. The strain distribution is nonuniform in the triaxial stress state, and the nonuniform strain distribution cannot satisfy the requirement of the extensometer. In this study, the finite element analysis software ABAQUS was used to analyze the strain distribution within the gauge length section of the specimen under compression. The finite element analysis uses a solid element. The constitutive model was the kinematic-hardening model and the specified value was selected for the material parameter from the standard CECS 410: 2015 [22]. The simulation results in Figure 2 show that when a −10% compressive strain was imposed on the small-gauge-length specimen, the strain was evenly distributed along the gauge length section, and the influence of the grip section and the fillet could be ignored. The strain distribution therefore satisfied the requirements of the extensometer.

**Figure 3:**
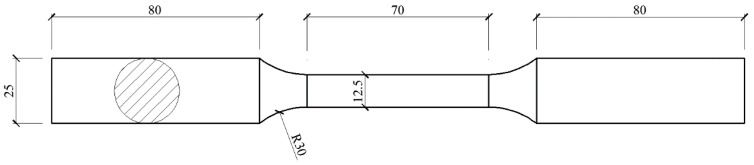
Conventional-gauge-length specimen.

Zhou et al. [20] suggested that the feasibility of small-gauge-length specimen can be verified by comparing the tensile test results of conventional-gauge-length specimens with those of small-gauge-length specimens. The tensile specimen was designed according to national design standard GB/T 228.1-2010 [23], as shown in Figure 3, in which the gauge length was 70 mm and gauge diameter was 12.5 mm.

**Figure 4:**
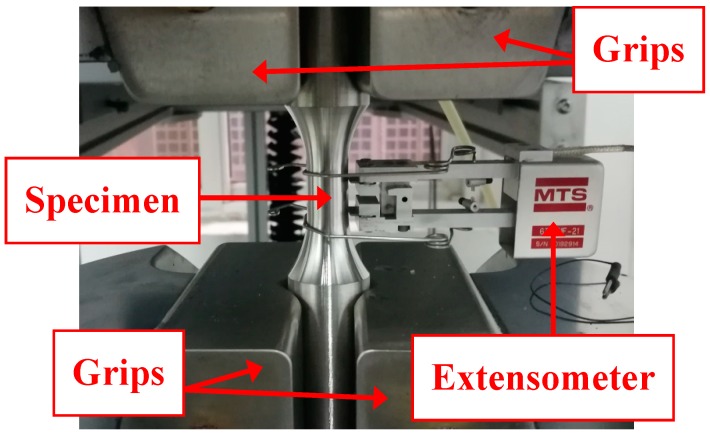
Arrangement of the extensometer.

The stainless steel plates were cut in the rolled direction. The test specimens were manufactured from stainless steel plates into round test coupons. The reduced section and round transition zone were machined using the numerically controlled equipment. The surfaces of the specimens were carefully polished using different grit sandpapers to eliminate surface defects. The test was performed on an MTS Landmark 370 hydraulic servo material testing machine with a maximum loading force of 500 kN. Computer-controlled loading procedures were used during the test. The test procedure complied with GB/T 7314-2005 [17]. During the test, the strains of the conventional-gauge-length specimens and small-gauge-length specimens were determined by an extensometer with a gauge length of 50 mm (strain measurement range −10% to +50%) and a gauge length of 10 mm (strain measurement range −10% to +20%), respectively. The extensometer models used were MTS 634.25F-24 and MTS 634.31F-21. The arrangement of the measuring device is shown in Figure 4. The force was measured by a force sensor of the testing machine and all data were recorded by a computer. When the stress was less than the nominal yield strength, the loading rate was 0.1 mm/min. When the stress was greater than the nominal yield strength, the loading rate was 0.5 mm/min. The recording frequency of the extensometer was 10 Hz.

When specimens are subjected to high non-elastic strains, the gauge length section has a radial contraction or expansion deformation, and the nominal stress and strain cannot accurately describe the “true” stress and strain states on the specimen [24]. In this study, the nominal stress and strain were converted into true stress and strain using Equations (1) and (2) [20]:

(1) $$\varepsilon_{\mathrm{true}}=\ln(1+\varepsilon_{\mathrm{nom}})$$

(2) $$\sigma_{\mathrm{true}}=\sigma_{\mathrm{nom}}(1+\varepsilon_{\mathrm{nom}})$$

In the formula, $\sigma_{\mathrm{true}}$ and $\varepsilon_{\mathrm{true}}$ are true stress and strain, respectively, and $\sigma_{\mathrm{nom}}$ and $\varepsilon_{\mathrm{nom}}$ are nominal stress and strain, respectively.

## 3. Results and Discussion

### 3.1. Comparison of Tensile Test Results

**Figure 5:**
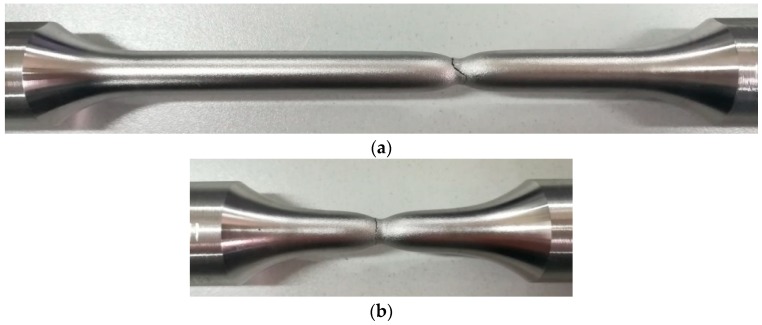
Tensile test failure morphology; (**a**) Conventional-gauge-length specimen; (**b**) Small-gauge-length specimen.

**Figure 6:**
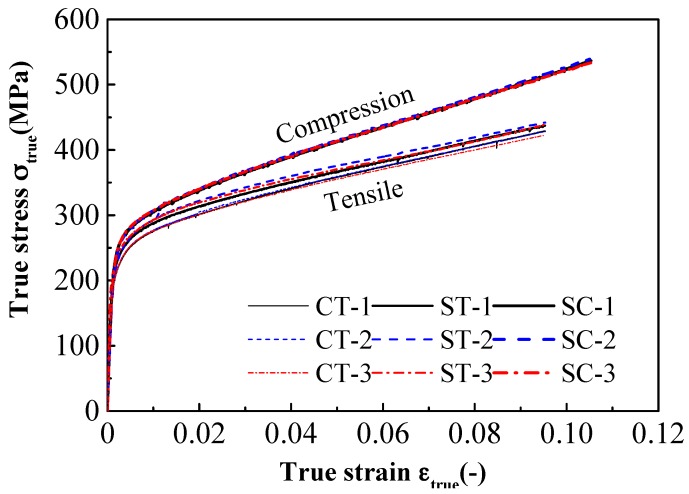
True stress-true strain curves for tensile and compression tests. (CT denotes tensile test of the conventional-gauge-length specimen, and ST and SC denote tensile test and compressive test of the small-gauge-length specimen).

Figure 5 shows photographs of the two specimens after fracture. The necking of the specimens is obvious before fracture. It shows that the two specimens had a large plastic deformation before fracture, which represents the true performance of the stainless steel. Figure 6 shows the true stress-true strain curves of the conventional-gauge-length specimen and the small-gauge-length specimen obtained from the tensile test. The 10% strain was consistent for both samples and the test curve of the small-gauge-length specimen was slightly higher than the conventional-gauge-length specimen’s test curve. This shows that the small-gauge-length specimen was uniformly deformed within the 10% strain of the gauge length section. Table 3 shows the nominal yield strength, tensile strength, and elongation after fracture of the two specimens. The measured strength values of the two test pieces were almost the same and the elongation after breaking was different. The elongation after fracture was inversely proportional to the length of the gauge length section; thus, the elongation after fracture of the small-gauge-length specimen was greater than that of the conventional-gauge-length specimen [25].

### 3.2. Compression Test

**Figure 7:**
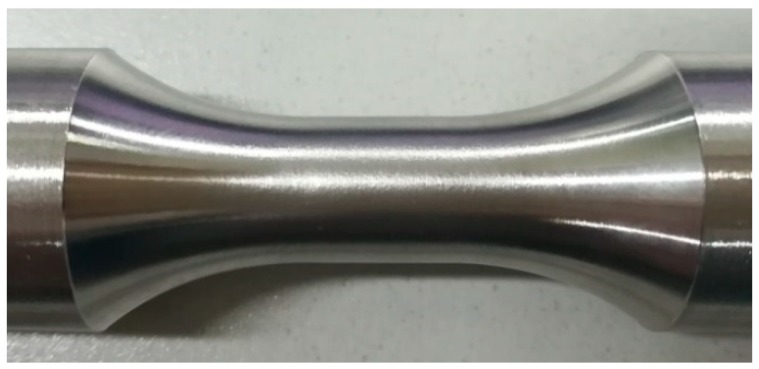
Specimen under −10% compressive strain.

Since the maximum measured value of the extensometer compressive strain is 10%, the test was terminated when the compressive strain reached 10% and no buckling occurred during the entire loading process. The specimen at the end of loading is shown in Figure 7. The gauge section of the specimen bulged under compression, and the diameter increased from 12.50 mm to 13.16 mm, or by 5.3%. The new type of the specimen with a small gauge length did not require the bracing jig and lubricating paste, as shown in Figure 4. This simplifies the compressive test preparation and reduces the testing cost. The compressive strain of the test can reach 10%.

**Figure 8:**
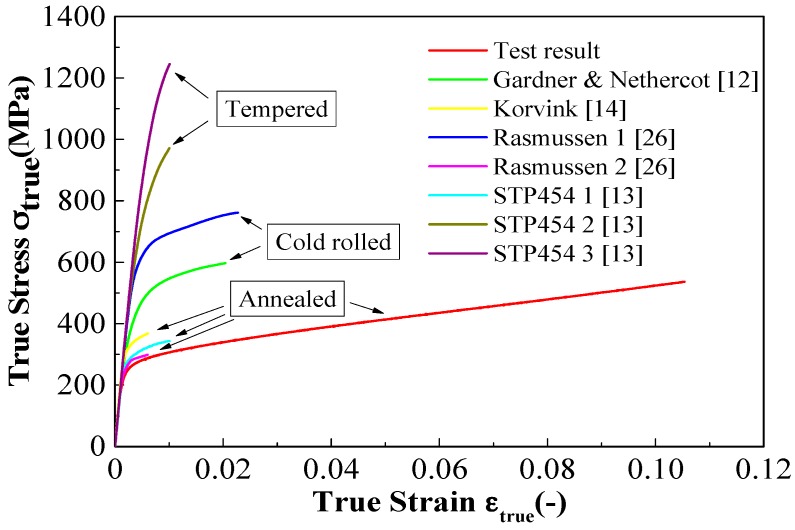
Comparison of compressive test results with the compressive test data in literature.

The strain-hardening behavior of the austenitic stainless steel in compression tests is affected by the manufacturing process. Considering the manufacturing process of the austenitic stainless steel, the published experimental results can be classified into three grades: annealed condition, tempered condition, and cold-formed condition. The material anisotropy is higher in the tempered condition and cold-formed condition [1,13]. We compared the mean stress-strain curve of the compressive test results with the published experimental results for the different manufacturing processes (Figure 8). The austenitic stainless steel adopted in this study was manufactured under the hot-rolled annealed condition. The results obtained under tempered and cold-formed conditions [12,14,26] are above the compressive stress-strain curve obtained in this study (Figure 8). In Figure 8, the compressive stress-strain curve is in agreement with the stress-strain curve under the annealed condition reported by Johnson and Kelsen [13] and Rasmussen et al. [26]. The test results represent the compressive behavior of austenitic stainless steel under the annealed condition.

The mechanical properties of compressive specimens are shown in Table 3. The tensile and compressive deformation modulus values (including initial modulus and secant modulus) were the same, as shown in Table 3. The true stress-true strain curves obtained by the compressive tests up to 10% strain are shown in Figure 6. There were significant differences in the strength values, as observed from the compression and tension test results. The proof stress values of the small-gauge-length specimens at 1% strain were 292 MPa and 316 MPa (i.e., difference of 8%) and at 10% strain, the values were 407 MPa and 601 MPa (difference of 48%) under tensile and compressive loading, respectively. The material anisotropy was found to be more remarkable at a higher level of imposed strain. This characteristic can be attributed to the strain-induced transformation phenomenon of austenite to martensite in stainless steel [8]. Since the formation of martensite is suppressed under tensile loading but is promoted under compressive loading [27,28], the proportion of martensite increases at high compressive strains, which enhances the strain hardenability of the austenitic stainless steel and postpones the onset of necking [8]. Therefore, the higher proportion of martensite caused by the high compressive loading is the main reason for the material anisotropy of stainless steel. In our study, when the true strain was 1%, the true stress-true strain curve of the tensile test and compression test of the small-gauge-length specimen began to separate, and with the increase in true strain, the difference in true stress between the two increased. When the true strain reached 10%, the true stress difference between the two exceeded 80 MPa (Figure 6). The comparison of the strength values measured in the compression test and tensile test of the small-gauge-length specimens, shown in Table 3, reveals that the nominal yield strength measured by the compression test of the small-gauge-length specimens was higher than the nominal yield strength measured by the tensile test of the conventional-gauge-length specimens. The elongation after fracture measured by the tensile test of the small-gauge-length specimens was much greater than that of the conventional-gauge-length specimens, and the difference in elongation after fracture between the conventional-gauge-length specimens and small-gauge-length specimens was due to the large difference in the ratio of the contraction segment to the standardization segment of the two specimens. The difference between the tensile and compressive stress-strain curves increased with increase of the strain. This is related to the different strain-hardening behavior being attributed to different microstructure evolution under the tensile and compressive loading.

### 3.3. Compressive Constitutive Model of Austenite Stainless Steel

#### 3.3.1. Constitutive Models

In the field of civil engineering, there are two constitutive models that describe the compressive properties of stainless steel: (1) Gardner and Nethercot revised model based on the Ramberg–Osgood model [12] and (2) Quach et al.’s three-stage model [11]. The modified model of Gardner and Nethercot is a combination of two Ramberg–Osgood models and is in good agreement with the stress-strain curve of stainless steel:

(3) $$\varepsilon =\left\{ \begin{array}{ll} \frac{\sigma }{E_{0}}+0.002{\left(\frac{\sigma }{\sigma_{0.2}}\right)}^{n} & \sigma \leq \sigma_{0.2}\\ \frac{\sigma -\sigma_{0.2}}{E_{0.2}}+\left(0.008+\left(\sigma_{1.0}-\sigma_{0.2}\right)\left(\frac{1}{E_{0}}-\frac{1}{E_{0.2}}\right)\right)\cdot {\left(\frac{\sigma -\sigma_{0.2}}{\sigma_{1.0}-\sigma_{0.2}}\right)}^{{n^{\prime }}_{0.2,1.0}}+\varepsilon_{0.2} & \sigma >\sigma_{0.2} \end{array}\right.$$

where $\sigma_{1.0}$ is the proof stress of 1.0% strain, n is the first curve’s hardening index, $\varepsilon_{0.2}$ is the total strain at stress $\sigma_{0.2}$, and ${n^{\prime }}_{0.2,1.0}$ is the second curve’s hardening index expressed by $\sigma_{0.2}$ and $\sigma_{1.0}$. Compared with the experimental data, the Gardner and Nethercot model has high accuracy when the compressive strain is less than 2% [11]. 

Olsson [29] conducted many experiments on uniaxial tensile and biaxial proportional loading and studied the plastic yield criterion and the hardening model of stainless steel. The stress-strain curves under uniaxial tension had two-stage strain hardening and the second stage strain hardening could be represented by a straight line [29]. Quach et al. [11] adopted Olsson’s conclusion and modified the Gardner and Nethercot model by adding a straight-line stage after the 2% proof stress *σ*_0.2_. The Quach model is in good agreement with the stress-strain curve of the compressive behavior (Equation (4)):

(4) $$\varepsilon =\{\begin{array}{cc} \frac{\sigma }{E_{0}}+0.002{\left(\frac{\sigma }{\sigma_{0.2}}\right)}^{n} & \sigma \leq \sigma_{0.2}\\ \frac{\sigma -\sigma_{0.2}}{E_{0.2}}+\left(0.008+\left(\sigma_{1.0}-\sigma_{0.2}\right)\left(\frac{1}{E_{0}}-\frac{1}{E_{0.2}}\right)\right)\cdot {\left(\frac{\sigma -\sigma_{0.2}}{\sigma_{1.0}-\sigma_{0.2}}\right)}^{{n^{\prime }}_{0.2,1.0}}+\varepsilon_{0.2} & \sigma_{0.2}<\sigma \leq \sigma_{2.0}\\ \frac{\sigma -a}{b\mp \sigma } & \sigma >\sigma_{2.0} \end{array}$$

where $\sigma_{2.0}$ is the proof stress of 2.0% strain, n is the first curve’s hardening index, $\varepsilon_{0.2}$ is the total strain at stress $\sigma_{0.2}$, ${n^{\prime }}_{0.2,1.0}$ is the second curve’s hardening index expressed by $\sigma_{0.2}$ and $\sigma_{1.0}$, and *a* and *b* are material constants. The model was verified by comparing the results with the compressive test results under ≤2.4% strain [11].

#### 3.3.2. Reliability of Constitutive Models

We fitted the Gardner and Nethercot model and the Quach model with the results of the compressive tests. The fitting parameters are shown in Table 4. The coefficients *R*^2^ of the regression equations were 0.988 and 0.997, respectively, suggesting that the two models perfectly correlate with the compressive test results.

**Figure 9:**
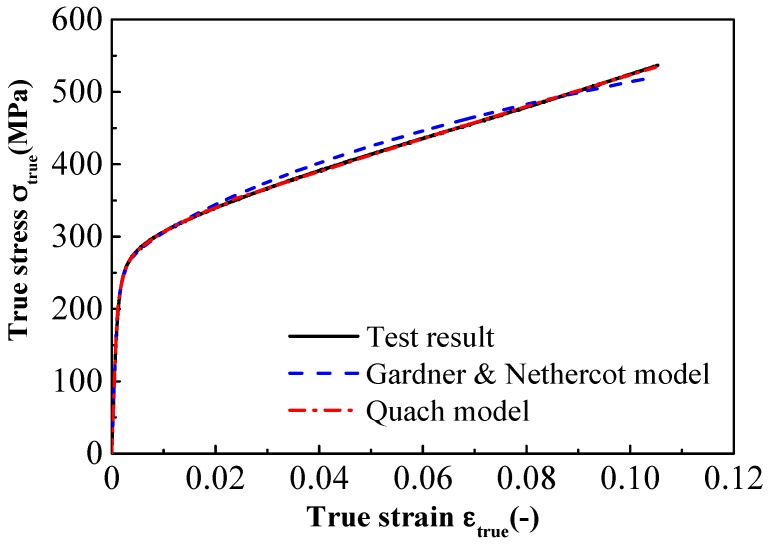
Comparison between the test and two fitting stress-strain curves.

The comparisons between model predictions and compressive experimental data are shown in Figure 9. The compressive stress-strain curve is the two-stage strain-hardening curve, wherein the second strain-hardening stage is represented by a straight line. This is the same as Olsson’s conclusion obtained from the experimental tensile data. The stress-strain curves predicted by these models coincide with the compressive experimental data. There is a slight difference between the predictions of the two models. The Quach model can accurately describe the second strain-hardening behavior, while the prediction curve of the Gardner and Nethercot model deviates from the compressive stress-strain curve. Therefore, the Quach model is in a better agreement with the test results than the Gardner and Nethercot model under high compressive strain.

## 4. Conclusions

Compressive mechanical properties and constitutive model of the austenitic stainless steel S30403 were studied. A new type of compressive specimen not aided by a bracing jig or a lubricating paste was then designed. A series of tensile and compressive tests were performed on austenitic stainless steel S30403 to obtain the stress-strain curves. The material anisotropy and strain-hardening behavior of austenitic stainless steel S30403 were also analyzed. In addition, the existing constitutive models of austenitic stainless steel were discussed by comparing them with the test results. The following conclusions can be drawn from the results:

(1) When the length-to-diameter ratio of the specimen is 1:1, the specimens do not buckle under high compressive strain of 10%. The small-gauge-length specimen with length-to-diameter ratio 1:1 is suitable for compression experiment in the high strain range.
(2) There is an obvious material anisotropy of the stress-strain response in the tension and compression of austenitic stainless steel under high strain conditions up to 10%. The compressive nominal yield strength is higher than the nominal tensile yield strength. The compressive initial Young’s modulus is close to the tensile initial Young’s modulus.
(3) The compressive stress-strain curve is a two-stage strain-hardening curve and the second strain-hardening stage is represented by a straight line. The Quach model agrees with the test results and can accurately describe the strain-hardening behavior of austenitic stainless steel under high compressive strains up to 10%.

## Figures and Tables

**Table 1:** Chemical composition specified by GB/T 4237-2015 (weight percentage).

w(C)	w(Si)	w(Mn)	w(P)	w(S)	w(Cr)	w(Ni)
0.03	0.75	2.00	0.045	0.030	17.50	8.00

**Table 2:** Mechanical properties specified by GB/T 4237-2015.

Grade	Nominal Yield Strength *σ*_0.2_/MPa	Tensile Strength *σ*_u_/MPa	Elongation after Fracture *ε*_u_/%	Longitudinal/Transverse Strain Reinforcement Coefficient n
S30403	180	485	40	6/8

**Table 3:** Test results.

Test No.	Initial Modulus *E*_0_/MPa	Secant Modulus *E*_0.2_/MPa	Nominal Yield Strength *σ*_0.2_^a^/MPa	Proof Stress of 1.0% Strain *σ*_1.0_^a^/MPa	Proof Stress of 10.0% Strain *σ*_10.0_^a^/MPa	Ultimate Strength *σ*_u_^a^/MPa	Elongation after Fracture *ε*_u_/%
CT-1	203,626	16,382	233	275	395	721	57
CT-2	194,100	14,981	236	278	396	715	56
CT-3	195,331	15,214	240	277	396	725	59
mean value	197,686	15,526	236	277	396	720	57
Standard deviation	4230	613	2.87	1.25	0.47	4.11	1.25
ST-1	194,689	15,596	242	290	406	720	94
ST-2	196,896	16,330	244	295	409	727	93
ST-3	195,460	15,784	245	292	407	723	92
mean value	195,682	15,903	244	292	407	723	93
Standard deviation	915	311	1.25	2.05	1.25	2.87	0.82
SC-1	206,405	15,196	267	317	600 ^b^	-	-
SC-2	195,471	16,014	267	317	604 ^b^	-	-
SC-3	203,590	17,046	269	314	600 ^b^	-	-
mean value	201,822	16,085	268	316	601 ^b^	-	-
Standard deviation	4636	757	0.94	1.41	1.89	-	-

^a^*σ*_0.2_, *σ*_1.0_, *σ*_10.0_ and *σ*_u_ are the nominal strength values; ^b^ For the description of compressive stress-strain behavior, there is no ultimate stress in compression due to the absence of the necking phenomenon, so we used *σ*_10.0_ instead of *σ*_u_.

**Table 4:** Compressive test fitting parameters using two models.

Model	*E*_0_/GPa	*σ*_0.2_/MPa	n	${n^{\prime }}_{0.2,1.0}$	a	b
G & N	202	268	8.5	1.5	-	-
Quach			8.5	1.7	296	2390
